# CircIFNGR2 enhances proliferation and migration of CRC and induces cetuximab resistance by indirectly targeting KRAS via sponging to MiR-30b

**DOI:** 10.1038/s41419-022-05536-8

**Published:** 2023-01-13

**Authors:** Qi Zhang, Yifeng Zheng, Jiajia Liu, Xiaoxiao Tang, Yuan Wang, Xianzheng Li, Huibin Li, Xiaoying Zhou, Shiru Tang, Yitao Tang, Xiaoyan Wang, Han He, Tingting Li

**Affiliations:** 1 Department of Pathology, Nanfang Hospital, Southern Medical University, Guangzhou, Guangdong China; 2 Department of Pathology, School of Basic Medical Sciences, Southern Medical University, Guangzhou, Guangdong China; 3 State Key Laboratory of Oncology in Southern China, Department of Experimental, Guangzhou, Guangdong China; 4 Medical Genetic Center, Guangdong Women and Children Hospital, Guangzhou, Guangdong China; 5 Department of Hematology, Nanfang Hospital, Southern Medical University, Guangzhou, Guangdong China

**Keywords:** Molecular biology, Cancer

## Abstract

Currently the clinical efficacy of colorectal cancer (CRC) which is the most common malignant tumors over the world has not reached an ideal level. Cetuximab, the monoclonal antibody targeting the extracellular domain of EGFR, has shown its great efficacy in the promotion of apoptosis and the inhibition of tumor cells-like characteristics in numerous cancers. However certain KRAS wild-type CRC patients unexpectedly show cetuximab resistance and the specific mechanism remains unclear. Circular RNAs (circRNAs) as the promising novel type of biomarkers in the cancer diagnosis and therapy, have been reported to be related with the drug resistance. In this study, with wondering the mechanism of cetuximab resistance in KRAS wild-type CRC patients, we evaluate the impact of circIFNGR2 on CRC and detect the association among circIFNGR2, miR-30b and KRAS via various experiments such as RT-qPCR, immunohistochemistry, luciferase assays, cell functional experiments and xenograft model. We conclude that circIFNGR2 induces cetuximab resistance in colorectal cancer cells by indirectly regulating target gene KRAS by sponging miR-30b at the post-transcriptional level. It is thus suggested that inhibition of circIFNGR2 can be a promising therapeutic strategy for malignant CRC patients with cetuximab resistance.

## Background

Currently, colorectal cancer (CRC) has been one of the most common malignant tumors over the world [1]. Clinically, the metastasis to distant organs in CRC progression occurs in nearly half of the CRC patients and the metastasis in CRC always seriously threatens human health, leading to unfavorable prognosis and high mortality [2–4]. Thus, there is an urgent need for more effective diagnosis and treatment strategies, in which precision medicine plays a paramount role. Analysis of the mechanism of occurrence, development, invasion, metastasis and recurrence in CRC at the molecular level may not only contributes to the exploration of novel molecular targets but also the new therapies with high efficacy.

Cetuximab exhibits promising efficacy in anti-CRC therapy, it has been applied as the first-line treatment of colon cancer, especially the metastatic CRC [5, 6]. Despite the great clinical efficacy of cetuximab such as the promotion of progression-free survival (PFS) and overall survival (OS), the improvement in the quality of life of patients and the low side effects, the benefits of cetuximab in CRC has been limited due to the chemotherapy resistance [7–9]. KRAS is the major oncogenic driver and leads to acquired therapy resistance in CRC [10–12]. Crystal study showed mutant KRAS and about 40% of wild-type KRAS in CRC patients were resistant to cetuximab [10, 12]. Thus, it is of great significance to explore the mechanism of cetuximab resistance in CRC patients, particularly at the molecular level, and to further search for molecular markers for effective cetuximab therapy.

Circular RNAs (circRNAs) are a class of endogenous long RNAs, characterized by the closed continuous loop without 5’–3’ polarity or the polyA tail [13]. Circular RNAs have once been regarded as the noncoding RNAs, actually in the latest few years their translational mechanism and the functions of the encoded peptides have been gradually revealed [13]. CircRNAs can act as microRNAs (miRNAs) sponges, interact with RNA binding proteins, modulate the stability of mRNAs and participate in the gene transcription and proteins translation [14–16]. In consideration of their conservation, abundance and tissue specificity, circRNAs may play roles as a novel type of molecular markers in some diseases, especially cancers and actually circRNAs have been reported in different types of cancer, including gastric cancer, breast cancer, CRC and hepatocellular carcinoma [17–20]. The journal *cell* has reported that circRNAs are correlated with the drug resistance. For instance, f-circM9 inhibits cancer cells from drug-induced apoptosis in acute promyelocytic leukemia [21], circRNA_101237 drives the cisplatin resistance in hepatocellular carcinoma [22] and circRNA_0025202 upregulates tamoxifen sensitivity and suppresses tumor progression via sponging miR-182-5p in breast cancer [23]. On the basic of these researches, we cannot help wondering whether certain circRNAs participate in cetuximab resistance among CRC patients.

In our study, we indeed found there were associations among circRNAs, miR-30b, KRAS and cetuximab resistance. We predicted that circIFNGR2 could sponged miR-30b via bioinformatic method and subsequently confirmed that circIFNGR2 promoted the proliferation and migration of CRC cells by targeting miR-30b/KRAS. In addition, we demonstrated that circIFNGR2 might act as a potential chemotherapy resistance marker in CRC.

## Method

### Tissue specimens and cell cultures

Fresh CRC specimens and the matched adjacent normal tissues were obtained from the Department of General Surgery, Nanfang Hospital. All tissues biopsies were frozen and preserved in liquid nitrogen. Patients’ medical records were collected to obtain the information such as age, gender, pathological stage, T stage, lymph node metastasis and distant metastasis.

Human colorectal cell lines FHC, LOVO, DLD1, SW480, HCT116, HCT8, HT29, SW620, CACO2, LS174T, and RKO, purchased from American Type Culture Collection Cell Biology Collection and maintained in the Department of Pathology Southern Medical University, were cultured in RPMI 1640(Invitrogen, Carlsbad, CA, USA) or DMEM (Sigma-Aldrich, St. Louis, MO, USA) containing 10% FBS (Invitrogen, Carlsbad, CA, USA) and 1% penicillin/streptomycin (Invitrogen, Carlsbad, CA, USA). The above cells were cultured at 37 °C with 5% CO_2_ [24, 25].

### RNA extraction and RT-qPCR

Total RNA from the tissues was extracted by the Trizol, chloroform, isopropanol, 75% ethanol and DEPC H_2_O. Total miRNA was extracted from the cells and tissues using the mirVana miRNA Isolation Kit (Ambion, Austin, TX, USA) referring to the manufacturer’s instructions. With the Evo M-MLV RT Mix Kit (ACCURATE BIOLOGY AG, Changsha, Hunan, China) and TaqMan miRNA Reverse Transcription Kit (ACCURATE BIOLOGY AG, Changsha, Hunan, China), the cDNA was then synthesized from total RNA and miRNA respectively. After the sufficient mixture of IQTM SYBR Green Supermix (ACCURATE BIOLOGY AG, Changsha, Hunan, China), 5 ng of cDNA and 10 pM of each primer, RT-qPCR was performed using the Applied Biosystems 7500 Sequence Detection system. The cycling conditions were set according to the previous study [26]. The data were normalized to the geometric mean of the reference gene GAPDH and calculated according to the 2-ΔΔCT formula. The forward and reverse sequences of the primers are shown in Additional file 1: Table S1.

### Wound healing assays

Cells in the logarithmic growth phase (5 × 10^5^ cells/well) were seeded into 6-well plates. A 100 μl pipette tip was used to scratch a monolayer in the middle of the well when the cell density reached about 90%. Keep the tip perpendicular to the bottom of the well to obtain an artificially straight wound. Remove and wash off the sloughed cells for three times a day. At the specific time point (0 h, 24 h, and 48 h), the wound in the same scratched line was observed and photographed. Each experiment was repeated for three times.

### Transwell chamber assays

Cells (1 × 10^4^/ chamber) were seeded into the upper Boyden chamber with an 8 μm pore size filter membrane. The transwell chambers were put in the 24-well plates with culture medium containing 10% fetal bovine serum as a chemoattractant. After 24 h, the cells on the upper filter were gently removed with a cotton swab. The cells that had migrated through the membrane were stained with hematoxylin for 10 min after the 4% paraformaldehyde fixation. The migrated cells were counted (10 random 200× fields per well) after washing the cells for three times to remove the paraformaldehyde and drying the membrane with a blower. Three independent experiments were conducted with data presented as the mean ± S.E.M.

### Colony formation assays

Cells were seeded in 6-well plates (5 × 10^2^ cells/well) for 2 weeks of culture with 10% FBS and 1% penicillin/streptomycin at 37 °C with 5% CO_2_. The plates were then washed with phosphate-buffered saline (PBS) for three times after the medium was removed. The cells were stained with hematoxylin for 20 min after the anhydrous ethanol fixation for 30 min. The colonies were defined as >50 cells/colony.

### Xenograft model in nude mice

#### Xenograft model was established as previous study described

For tumorigenesis assays, female BALB/c athymic nude mice (4–6 weeks of age, 18–20 g) were obtained from the Animal Center of Southern Medical University, Guangzhou, China) and 2 × 10^6^ cells per mouse were hypodermic injected into these mice. These mice were sacrificed at about 7 to 8 weeks. After dissection and resection, the tumor was taken out and then placed in 10% neutral formalin buffer for 24 h. The mice were raised with specific pathogen-free conditions. All protocols in this study complied with the Use Committee for Animal Care and were executed according to institutional guidelines which passed through strict deliberation. The tumor size was measured with a vernier caliper and the tumor volume was calculated according to the following formula: 0.5 × A × B^2^, in which the character A was the diameter of the base of the tumor and the character B was the corresponding perpendicular value. As for the metastasis experiment [27–29], 1 × 10^6^ cells suspended in 100 μL phosphate‐buffered saline (PBS) were injected to the subcapsular of the spleen of the nude mice to establish the spleen-liver metastasis and 1 × 10^6^ cells suspended in 100 μL PBS were injected into the nude mice tail vein to establish the lung-metastasis models. At 5 weeks after injection, live images were captured by MRI and systemic organs were immediately resected for ex vivo evidence of metastatic signals.

### Immunohistochemistry

The tissues were embedded in paraffin, cut into 4 μm-thick sections, baked at 65 °C for 50 min, and then dewaxed by dimethylbenzene and alcohol. After dewaxing with xylene, to decrease the activity of endogenous peroxidase, the sections were treated with 3% hydrogen peroxide. Antigen retrieval and the block of nonspecific binding were conducted in order via soaking the sections in citrate buffer and incubated the sections with 1% bovine serum albumin (BSA). Primary antibodies against Ki-67 (1:500; Servicebio, Wuhan, China), KRAS (1:200; Cell Signaling Technology, Danvers, MA, USA), and an appropriate secondary antibody (Zhongshan Golden Bridge, Beijing, China) were used according to the manufacturer’s instructions. DAB and hematoxylin were used to incubation of the sections, which were subsequently scored according to both the proportion of tumor cells with positively staining and the intensity of staining by three observers individually.

### Luciferase assays

Inoculate CRC cells in triplicate (3 × 10^5^ cells/well) into 24-well plate, and then culture the cells for 24 h until the cells adhere to the plate. PmirGLO-basic was used as a vector to construct a wild-type plasmid and a mutant plasmid of miR-30b. Lipofectamine 3000 Reagent (Invitrogen) were used in the transfection with constructed pmirGLO-basic luciferase reporter plasmid (1.5 μg, WZ Biosciences) or pmirGLO-basic control luciferase plasmid (1.5 μg, WZ Biosciences). According to the manufacturer’s instructions, the dual-luciferase reporter assay system (Promega) was used to examine relative luciferase and Renilla activities 30 h after the transfection. Experiments were all carried out for at least 3 times, and the data were expressed as mean ± SD.

### Flow cytometry assay

After incubation with 5 μg/ml cetuximab (Macklin, 205923-56-4) or DMSO respectively for 36 h, the indicated CRC cells were placed in a 1.5 ml tube, washed twice with PBS and centrifuged at 400 x *g* for 2 min. After retaining the sediment, 60 μl of surface staining buffer (PBS, pH7.4, 0.1% BSA) mixed with antibodies against CD44 and CD133 at 1 μg/ml (BD Pharmingen, Franklin Lakes, NJ, USA) was used to resuspend the cells which were subsequently incubated for 30 min at 4 °C and then resuspended in PBS without washing and discarding the PBS. FACS flow cytometer and FlowJo software were used to perform the analysis.

### Cell Counting Kit-8 (CCK8) assay

CCK8 assay was performed according to the manufacturer’s instructions (Yeasen biotech, Shanghai, China). Indicated cells were seeded into the 96-well plate and respectively incubated for 1, 2, 3, 4, 5, and 6 days at 37 °C with 5% CO_2_. 10 μl CCK8 solution was added to the indicated wells and subsequently the cells were incubated for 2 h at 37 °C with 5% CO_2_. The absorbance values of wells were measured with the microplate detector (BioTek Epoch, USA) at 450 nm. Experiments were all carried out for at least 3 times.

### Western blotting assay

Lyse the cells were lysed to obtain cellular proteins samples. After 10% SDS-PAGE, the separated protein samples were transferred onto the polyvinylidene difluoride (PVDF) membranes (Millipore, Billerica, MA, USA). 5% skim milk was used to block the nonspecific antigens for 2 h. The membranes were reacted with primary and secondary antibodies. Band exposure and analyses were finally conducted. The anti-KRAS (Cell Signaling Technology, Danvers, MA, USA) and the anti-GAPDH monoclonal antibody (Bioworld technology, co, Ltd, Nanjing China) served as an internal reference were used as the primary antibodies; the Goat anti-Rabbit IgG-HRP (Bioworld technology, co, Ltd, Nanjing China) served as the secondary antibody.

### Fluorescence in situ hybridization (FISH)

The CY3-labeled probes specific to circIFNGR2 and FITC-labeled probes against miR-30b (Additional file 1: Table S1) were prepared by Geneseed Biotech, Guangzhou, China. Cells were cultured and fixed with 4% paraformaldehyde in PBS for 30 min on confocal dishes and then treated with PBS containing 0.5% Triton X-100. Dilute the probes in PCR tubes with pre-hybridization solution (Ribobio, Guangzhou, China), then heat the tubes in a PCR block at 85 °C for 5 min. Incubate the tubes at 37 °C for 2 min and then discard the pre-hybridization solution. Diluted probes with the volume of 100 μl/section was added to the samples, glass coverslips were used to cover the samples and rubber cement was used to seal the samples. After incubation overnight in a humidified hybridization chamber at 37 °C, the confocal dishes were washed three times with the washing buffer 4X SSC, 2X SCC and 1X SCC at the same temperature, then stained with and DAPI. Finally, the images were obtained on Inverted laser confocal microscope FV3000 (Olympus Global Homepage, Japan).

### RNA pull-down assays

According to the manufacturer’s protocol, Pierce^TM^ was used for RNA pulldown assays Magnetic RNA-Protein Pull-Down Kit (Thermo Scientific, MA, USA). The probe was used for incubation with magnetic beads at 25 °C for 2 h to generate probe-coated beads. Cells were washed by PBS for twice and then incubated with the RIP lysates for 2 h at 25 °C. Cell lysate and probe were incubated together at 4 °C overnight. After washed with wash buffer containing proteinase K for 1 h at 25 °C, the RNA mix bound to the beads was eluted and extracted. Then the complexes in the pull-down were analyzed via RT-qPCR.

### RNA immunoprecipitation

RNA immunoprecipitation (RIP) assay was conducted by RiboCluster Profiler RIP-Assay Kit (MBL Beijing Biotech Co, Ltd, China) according to the manufacturer’s protocol. The experiments were performed in cells transiently overexpressing miR-30b or miR-NC.

Cells were harvested by trypsinization, resuspended in PBS, lysed in 100% RIP Lysis Buffer with proteinase and RNase inhibitors. Then the RIP lysate was incubated with RIP buffer containing magnetic beads conjugated with human anti-Ago2 antibody (MBL Beijing Biotech Co, Ltd, China) or nonspecific mouse IgG antibody (Cell Signaling Technology, USA). After 24 h, the mixture containing RNA was washed five times and resuspended in buffer containing RNase-free DNase and proteinase K. Then total RNA was extracted from the eluent and analyzed via RT-qPCR.

### Cetuximab treatment experiments

For the cetuximab treatment assay in vitro, female BALB/c athymic nude mice (4–6 weeks of age, 18–20 g) were obtained from the Animal Center of Southern Medical University, Guangzhou, China. 2 × 10^6^ tumor cells per mouse were subcutaneously inoculated into the above mice. When the tumor volume reached approximately 150 mm^3^, the tumor-bearing mice were randomly grouped. Mice in the two main groups were intraperitoneally injected with 20 mg/kg cetuximab (Macklin, 205923-56-4) or DMSO three times per week and the tumors were monitored every 3 days and their diameters (a, cm) and widths (b, cm) were measured twice a week. The approximate volume of tumors (V, cm^3^) was calculated based on the formula: V = (a × b^2^)/2. 50 days later, the tumors were resected, placed in 10% neutral buffered formalin for 24 h and then used for immunochemistry experiment.

For a series of functional assays in vivo mentioned above, cells were treated with the four different conditions as follows. Cells in the first group with no circIFNGR2 transfection were treated with DMSO, cells in the second group with no circIFNGR2 transfection were treated with cetuximab, cells in the third group transfected with circIFNGR2 were treated with DMSO and cells in the fourth group transfected with circIFNGR2 were treated with cetuximab. Then CCK8, transwell chamber, colony formation and flow cytometry assays were performed as previous protocols.

### Statistical analyses

All plotted and counted data and relative statistical analyses were performed by using SPSS19.0 for Windows, presented as mean ± SD. Any *p* < 0.05 was considered to be statistically significant. The difference between groups was accessed by the student *t*‐test and the χ^2^ test. The difference in the miR-30b or hsa_circIFNGR2 expression level between carcinomatous and normal CRC tissues was evaluated by a paired *t* test. Clinical pathological characteristics of circ_ IFNGR2 expression in CRC patients were analyzed by a two-sample *t* test. The linear relationship between circ_ IFNGR2 and miR-30b expression levels in colorectal cancer cells was measured by Pearson correlation coefficient.

## Results

### Upregulated circIFNGR2 was correlated with the occurrence of colorectal cancer

**Fig. 1:**
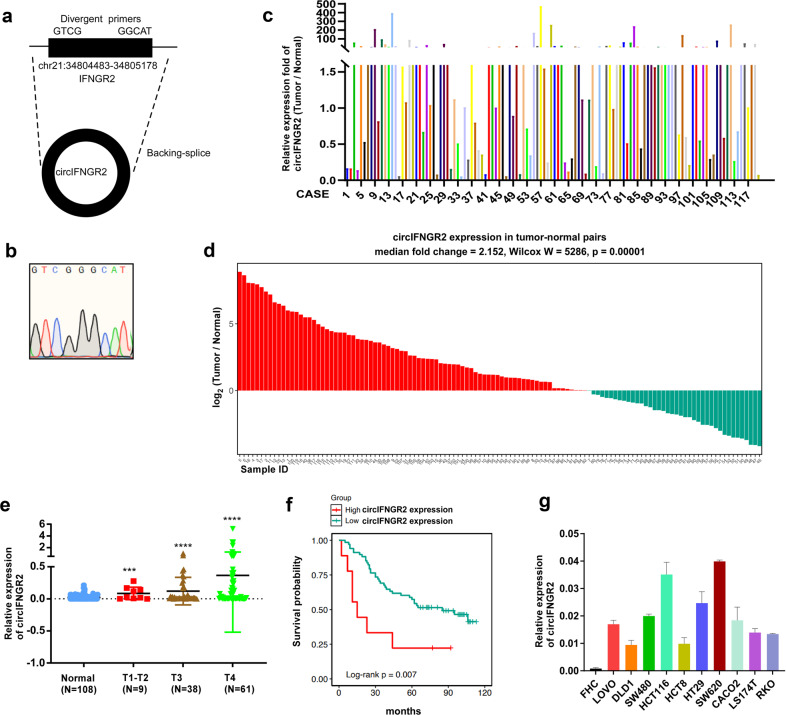
circIFNGR2 is overexpressed in colorectal cancer. **a** circIFNGR2 is generated by splicing the INFGR2 gene, located on chromosome. **b** The back-splice junction sequences of circIFNGR2 shown by Sanger sequencing results. **c** RT-qPCR of circIFNGR2 in 120 paired human CRC tissues and the matched adjacent normal tissues. **d** The relative expression of circIFNGR2 in 120 normal and cancerous tissues was detected by Wilcoxon test. **e** The correlation between the expression of circIFNGR2 and clinical stage (*p < 0.05, **p < 0.01). **f** Kaplan–Meier survival analysis of WT-KRAS CRC patients with low and high expression of circIFNGR2. **g** circIFNGR2 expression was determined using RT-qPCR analysis in 10 colorectal cancer cell lines and FHC cell line.

Expression of circIFNGR2 was detected in CRC (Fig. 1).

Hsa_circIFNGR2 (hsa_circ_001185, hsa_circ_000339), with the genomic length of 695 bp, is located at chr21:34804483-34805178 (Fig. 1a) and its back-splice junction sequences is shown in Fig. 1b. To assess the correlation between circIFNGR2 and CRC, we examined circIFNGR2 expression in 120 pairs of wild type (WT)-KRAS CRC biopsies and their adjacent normal tissues by RT-qPCR. Results revealed that compared to the normal tissues, nearly 59.2% (71/120) of the paired tumor tissues showed the significantly higher expression (T/N > 1.5-fold) of circIFNGR2 (*P* < 0.05, Fig. 1c). RT-qPCR of mutant (MUT)-KRAS CRC biopsies and their adjacent normal tissues showed that nearly 67.69% (44/65) of the paired tumor tissues showed the significantly higher expression (T/N > 1.5-fold) of circIFNGR2 (*P* < 0.05, Additional file 5: Fig. S4a). There was no significant difference in circIFNGR2 expression between WT-KRAS and MUT-KRAS CRC (Additional file 5: Fig. S4b). Simultaneously, the Wilcoxon test demonstrated that the circIFNGR2 expression level from the CRC tissues samples was markedly higher than that from adjacent normal tissues (Fig. 1d). Statistical distribution of circIFNGR2 in the CRC patients were analyzed on the basis of sex, clinical T stage and histological classification (Table 1). The results demonstrated circIFNGR2 upregulation was significantly correlated with the tumor TNM stage of CRC while its expression was almost unrelated to the other classified indexes. Besides, student *t* test also revealed that the expression of circIFNGR2 was correlated with the clinical T stage. As is shown in Fig. 1e, circIFNGR2 expression level was relatively low in the normal tissues, while its expression level was higher with the progression of the tumors and was remarkably upregulated in T4 stage. Moreover, Kaplan–Meier survival analysis showed that high expression of circIFNGR2 tightly related to a poor prognosis of WT-KRAS CRC (Fig. 1f) and MUT-KRAS CRC (Additional file 5: Fig. S4c). Next, RT-qPCR was applied in 11 CRC cell lines to evaluate the expression of circIFNGR2, including FHC, LOVO, DLD1, SW480, HCT116, HCT8, HT29, SW620, CACO2, LS174T, and RKO (Fig. 1g). The WT- KRAS cell lines such as CACO2 and HCT8 together with the MUT- KRAS cell lines such as SW480 and HCT116 were selected for most of the following experiments.

**Table 1:** Correlation between clinicopathological features and circIFNGR2 expression in 108 CRC tissues.

Characteristics	circIFNGR2 expression		*P*
	Low	High	
Gender			
Male	28	22	0.2468
Female	25	33	
Age			
*n* > =mean (56)	33	24	0.1227
*n* < mean (56)	21	30	
Differentiation			
Orrelation between clinicopWell	19	20	>0.9999
Orrelation between clinicopModerate - poor	33	36	
T classification			
1–2	2	7	0.0073
3–4	69	30	
*N* classification			
0	22	40	0.0401
>0	24	22	
Distant metastasis			
No	44	32	0.0082
Yes	27	5	

### Overexpression of circIFNGR2 enhances proliferation, invasion and migration capacities of CRC cells

**Fig. 2:**
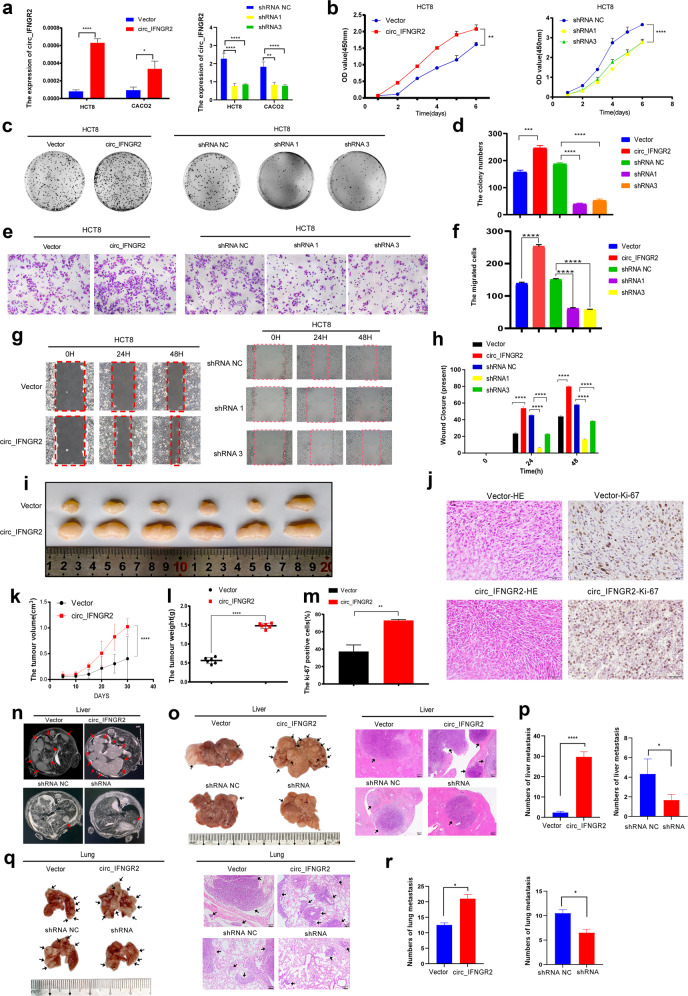
Overexpression of circIFNGR2 promoted proliferation, invasion and migration capacities of CRC cell. **a** The transfection efficiency of circIFNGR2 (named as circIFNGR2), circIFNGR2-shRNA1 (named as shRNA1) and circIFNGR2-shRNA3 (named as shRNA3) in HCT8 and CACO2 cells was analyzed by RT-qPCR. **b** CCK8 assays manifested the positive effect of circIFNGR2 on CRC cells growth **c** Representative image of colony formation assay in HCT8 cells. **d** Statistical analysis of the results of cell colony formation assays. **e** Representative images of transwell chamber assays in HCT8 cells. **f** Statistical analysis of the number of invasive cells in the transwell chamber assay. **g** Wound healing assay results showed the migration of HCT8 cells at 3 specific time intervals. **h** Statistical analysis of wound healing assay results. **i** Images of the tumor xenograft model. **j** Hematoxylin and Eosin (H&E) and Ki-67 immunohistochemical analysis on subcutaneous tumors from mouse tumor xenograft models. **k** Comparison of tumor volume of indicated cells. **l** Comparison of tumor weight of indicated cells. **m** Statistical analysis of Ki-67 positive cells. The error bars represent the means ± SDs from three independent experiments. *p < 0.05, **p < 0.01, ***p < 0.005. **n** Images of the mice spleen-liver metastasis model captured by MRI. **o** Images of spleen-liver metastasis tumor (left) and corresponding HE staining (right). **p** Statistical analysis of the number of the spleen-liver metastasis tumors. **q** Images of tail vein-lung metastasis tumor (left) and corresponding HE staining (right). **r** Statistical analysis of the number of the tail vein-lung metastasis tumors.

Next, the effect of overexpression of circIFNGR2 on proliferation, invasion, and migration capacities of CRC cells was tested (Fig. 2).

Using RT-qPCR to analyze and measure the expression of circIFNGR2, we confirmed that hsa-circIFNGR2 mimic oligonucleotides were successfully transfected into the WT-KRAS cell lines (CACO2 and HCT8, Fig. 2a, right) and MUT- KRAS cell lines (SW480 and HCT116, Additional file 5: Fig. S4d) and circIFNGR2 inhibitors were also successfully expressed in above cell lines (Fig. 2a, left). The following cell function assays revealed the influence of circIFNGR2 on CRC cells. CCK8 (Fig. 2b) and colony formation (Fig. 2c, d) assays showed overexpression of circIFNGR2 increased proliferation ability of HCT8 cells, while inhibition of circIFNGR2 suppressed their proliferation ability. Subsequently, transwell chamber (Fig. 2e, f) and wound healing assays (Fig. 2g, h) respectively verified that overexpression of circIFNGR2 significantly enhanced the invasion and migration capacities of HCT8 cells. The above cell function assays were repeated in CACO2, and the same results as expected were shown in Additional file 2: Fig. S1a–g.

We next investigated whether circIFNGR2 can affect the function of CRC cells in vivo and the results were surprisingly consistent with above in vitro function assays. CRC cells stably overexpressing circIFNGR2 were subcutaneously injected into six nude mice. The result of the CRC xenograft models (Fig. 2i) revealed that the volumes of the tumors formed by circIFNGR2-overexpressing CRC cells shown distinctly faster growth than those formed by control cells in terms of tumor volume (Fig. 2k) and weight (Fig. 2l). On top of that, HE staining of the subcutaneous tumors (Fig. 2j, left) shown lager cells quantity in the circIFNGR2 group than in the control group and the percentage of Ki67-positive cells (Fig. 2j, right) in subcutaneous tumors was higher in the circIFNGR2 group than in the control group. In addition, the circIFNGR2 inhibition group significantly showed the smaller volume, lower weight and less Ki67-positive cells of subcutaneous tumors compared to control group (Additional file 2: Fig.S1h–j). In the metastasis experiments, the spleen-liver metastasis model together with its MRI images (Fig. 2n) demonstrated that overexpression of circIFNGR2 significantly increased the number of the CRC cell metastatic nodules in the mice liver (Fig. 2o, p) and tail vein-lung metastasis models revealed that overexpression of circIFNGR2 remarkedly increased the number of the CRC cell metastatic nodules in the mice lung. (Fig. 2q, r).

Taken together, these data demonstrated a remarkable role of circIFNGR2 in the proliferation, invasion, and migration capacities of CRC cells.

### circIFNGR2 affects the expression of WT-KRAS and the downstream signaling

circIFNGR2 increased the mRNA levels of both WT-KRAS (Additional file 6: Fig. S5a) and MUT-KRAS in CRC cells (Additional file 6: Fig. S5b). Furthermore, we subsequently performed western blotting (Additional file 6: Fig. S5c, d) to detect the protein expression level of the corresponding KRAS downstream signaling proteins such as p-ERK and p-AKT. Interestingly, results showed that the protein expression level of p-ERK and p-AKT was upregulated in the circIFNGR2 overexpressed group of WT-KRAS cells, while the protein expression level of p-ERK and p-AKT showed little difference in the MUT-KRAS CRC cells groups. These results aroused our interests for it was known that KRAS can promote cell proliferation and inhibit the apoptosis of cells via activating the downstream AKT signaling. In the WT-KRAS CRC patients, circ_ IFNGR2 could upregulate the expression of KRAS, subsequently activating the downstream AKT signaling and promoting the cetuximab resistance of CRC cells. However, in the MUT-KRAS CRC patients, the downstream signaling of KRAS is persistently activated and thus circ_ IFNGR2 played a weak role in cetuximab resistance of CRC cells. Thus, our subsequent research was mainly focus on the relationship between circIFNGR2 and cetuximab resistance in the WT-KRAS CRC patients.

### circIFNGR2 sponges miR-30b to inhibit the transcriptional regulatory activity of miR-30b

**Fig. 3:**
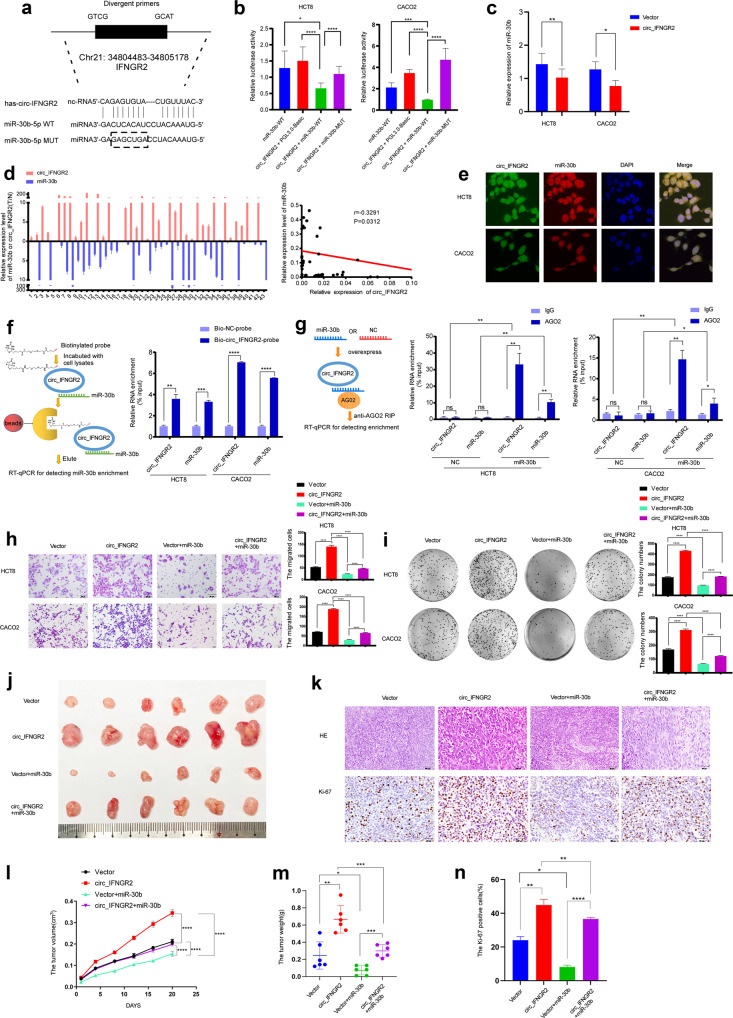
circIFNGR2 directly targets the miR-30b to suppress its transcriptional regulatory activity. **a** The predicted binding sites of circIFNGR2 and miR-30b. **b** circIFNGR2 affects the luciferase activity of miR-30b 3’UTRs in HCT8 and CACO2 cells. **c** The relative expression of miR-30b in HCT8 and CACO2 cells overexpressing circIFNGR2. **d** RT-qPCR analysis of circIFNGR2 and miR-30b expression in 43 human CRC tissues (left); correlation analysis indicated that the expression of circIFNGR2 is negatively correlated with miR-30b expression (right). **e** Fluorescence in situ hybridization confirmed the co-location of miR-30b (green) and circIFNGR2 (red) in HCT8 and CACO2 cells. Cell nucleuses were counterstained with DAPI (blue). **f** Graphical RNA pull-down flow chart (left); whole cell lysates from HCT8 and CACO2 cells were incubated with biotinylated probes against circIFNGR2 or NC; endogenous circIFNGR2 and miR-30b enrichments were examined by RT-qPCR (right). **g** Graphical anti-Ago2 RNA immunoprecipitation flow chart (left); the complex containing circIFNGR2 and miR-30b was captured, circIFNGR2 and miR-30b enrichment were detected via RT-qPCR (right). **h** Representative images of transwell chamber assay in indicated HCT8 and CACO2 cells (left); statistical analysis of the migrated cells (right). **i** Representative images of colony formation assay in indicated HCT8 and CACO2 cells (left); statistical analysis of the number of colony (right). **j** Image of the subcutaneous tumor mode. HCT-8-vector, HCT-8-circIFNGR2, HCT-8-vector + miR-30b and HCT-8-circIFNGR2 + miR-30b cells were subcutaneously injected into nude mice separately (*n* = 6/group). **k** H&E and Ki-67 immunohistochemistry assays of tumor sections. **l** The tumor volume of the four designated groups measured at designated time. **m** Statistical analysis of tumor weight in four indicated groups. **n** Statistical analysis of Ki-67 positive cells in tumor tissues. The error bars represent the means ± SDs from three independent experiments. *p < 0.05, **p < 0.01, ***p < 0.005.

Bioinformatics website starBase [30] was applied to predict that circ _IFNGR2 could bind to miR-30b. This inspired us to further explore the interaction between circIFNGR2 and miR-30b (Fig. 3). The predicted complementary sites between circIFNGR2 and miR-30b were shown in Fig. 3a. According to the complementary sites, we then constructed the mutant fragments of miR-30b (Fig. 3a). We then subcloned the wild-type and mutant fragments of miR-30b separately into the pmirGLO-basic luciferase reporter vectors. As is shown in Fig. 3b, in comparison with the other control groups, the luciferase activity in cells co-transfected with miR-30b-WT vector was significantly reduced by the overexpression of circIFNGR2 while the luciferase activity of cells co-transfected with miR-30b-MUT vector was nearly not influenced by circIFNGR2. Besides, RT-qPCR analysis revealed that the expression of miR-30b in HCT8 and CACO2 cells was significantly downregulated with circIFNGR2 overexpressed (Fig. 3c). Apart from the above, we detected the expression levels of circIFNGR2 and miR-30b in 43 fresh colorectal cancer tissues via RT-qPCR and results illustrated that circIFNGR2 was markedly negatively correlated with miR-30b (*r* = 0.3291, *p* = 0.0312, Fig. 3d). To further detect the relationship between circIFNGR2 and miR-30b, we conducted FISH assay and the results revealed the apparent co-localization of circIFNGR2 and miR-30b in cytoplasm (Fig. 3e). Moreover, the RNA pull-down experiment proved that the biotinylated probe targeting circIFNGR2 could simultaneously pull down miR-30b in HCT8 and CACO2 cells (Fig. 3f). RIP result demonstrated that circIFNGR2 pulled down by anti-Ago2 targeting miR-30b was significantly enriched in cells overexpressing miR-30b (Fig. 3g). The above RNA pull-down and RIP assays further contributed to validation of the direct binding of circIFNGR2 and miR-30b.

Given that miR-30b functioned as a tumor suppressor gene in CRC, we next preformed a series of cell functions assays to explore whether miR-30b could reverse the effect of circIFNGR2 on CRC cells functions. As expected, transwell chamber and colony formation assays showed that miR-30b dramatically suppressed the inhibiting effect of circIFNGR2 on invasion and proliferation capacities in CRC cells (Fig. 3h, i). Besides, CCK8 and wound healing assays (Additional file 3: Fig. S2a, b) further confirmed that miR-30b inhibited the pro-tumor ability of circIFNGR2. The co-transfection of miR-30b and circIFNGR2 strongly promoted the apoptosis of HCT8 and CACO2 cells compared with the group transfected with circIFNGR2 (Additional file 3: Fig. S2c).

To further explore whether miR-30b could inhibit pro-tumor effect of circIFNGR2 in vivo, we divided 24 nude mice into four groups and subcutaneously injected HCT8/vector, HCT8/circIFNGR2, HCT8/vector + miR-30b and HCT8/circIFNGR2 + miR-30b cells respectively. As is shown in Fig. 3j–n, the subcutaneous tumors of the nude mice injected with the HCT8/ circIFNGR2+ miR-30b group showed the smaller volume, smaller weight and less Ki-67-positive cells, compared to the HCT8/circIFNGR2 group.

Taken together, these results demonstrated that circIFNGR2 can affected the function of CRC cells by targeting miR-30b both in vivo and in vitro.

### circIFNGR2 affects the expression of KRAS by targeting miR-30b

**Fig. 4:**
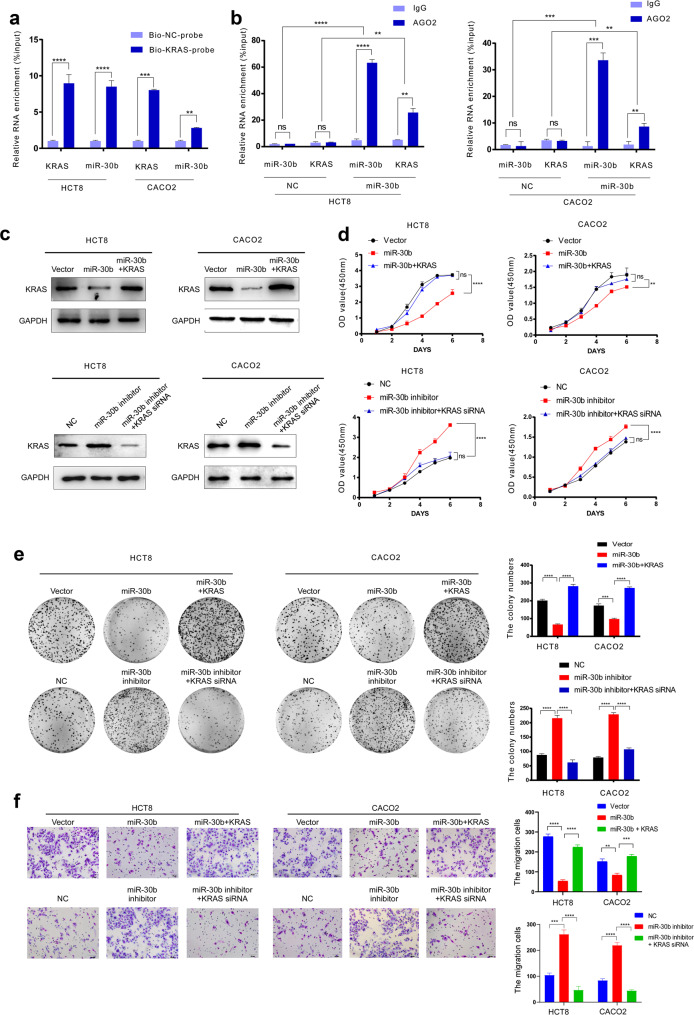
MiR-30b targets KRAS to suppress the expression of KRAS and inhibits cellular progressions in CRC. **a** Incubated whole-cell lysates of HCT8 and CACO2 cells with biotinylated probes specific to KRAS or NC, then RT-qPCR was executed to detect the enrichment of endogenous cir_IFNGR2 and KRAS. The results were expressed as the percentage of pull-down to the input. **b** Anti-Ago2 were immunoprecipitated with the complex containing miR-30b and KRAS in HCT8 (left) and CACO2 (right) cells using RIP, and then subjected to RT-qPCR analysis to detect the enrichment of miR-30b and KRAS. All tests were performed in triplicate. **c** Western blotting was performed to determine the protein expression of KRAS in the indicated cells (left). **d** CCK8 assay revealed the cell growth ability of the indicated cells **e** Representative image of colony formation assay in HCT8 and CACO2 cells(left), Statistical analysis of results(right). **f** Representative image of transwell chamber experiment in HCT8 and CACO2 cells (left), Statistical analysis of result s(right). The error bars represent the means ± SDs from three independent experiments. *p < 0.05, **p < 0.01, ***p < 0.005.

**Fig. 5:**
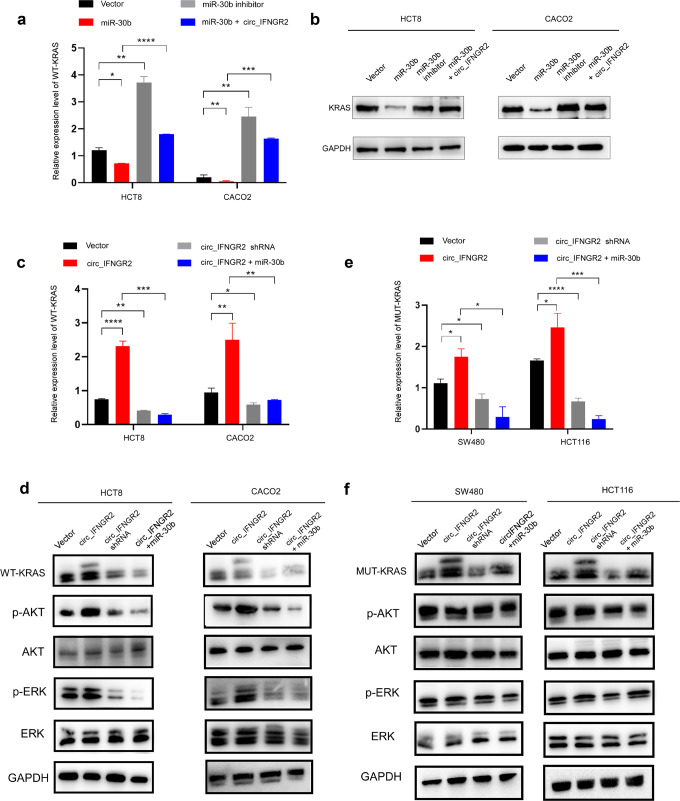
circIFNGR2 sponges miR-30b to reduce KRAS expression. **a** RT-qPCR was used to detect the relative expression level of WT-KRAS in the indicated cells. **b** Western blotting was performed to determine the protein expression of WT-KRAS in the indicated cells **c**, **e** RT-qPCR was used to detect the relative expression level of WT-KRAS in the indicated cells. **d**, **f** Western blotting was performed to determine the protein expression of KRAS and its downstream proteins. The error bars represent the means ± SDs from three independent experiments. *p < 0.05, **p < 0.01, ***p < 0.005.

In previous studies, we have confirmed that miR-30b directly targets KRAS. In this study, we further verified the relationship among circIFNGR2, miR-30b and KRAS (Figs. 4 and 5). The results showed that endogenous miR-30b was pulled down by biotin-labeled probes targeting KRAS (Fig. 4a). Anti-Ago2 RIP assay preformed in HCT8 and CACO2 cells showed that there was a large enrichment of KRAS pulled down by anti-Ago2 against miR-30b (Fig. 4b).

By co-transfecting miR-30b and KRAS in HCT8 and CACO2 cells, western blotting, CCK8 and colony formation assays revealed that compared with the control group, the expression level of KRAS protein, the proliferation ability of CRC cells and the number of colonies in the co-transfection group were significantly increased (Fig. 4c–e). Transwell chamber assays showed that the invasion ability of CRC cells was enhanced greatly after co-transfection compared with the control group (Fig. 4f). However, while silencing miR-30b and KRAS together, the opposite results occurred (Fig. 4c–f). Subsequent RT-qPCR and western blotting assays revealed that the expression level of KRAS was downregulated by overexpression of miR-30b and upregulated by inhibition of miR-30b (Fig. 5a, b). However, transfection with circIFNGR2 in the CRC cells reversed the KRAS downregulation (Fig. 5a, b). Besides, circIFNGR2 increased the expression of both WT-KRAS (Fig. 5c) and MUT-KRAS in CRC cells (Fig. 5e). Conversely miR-30b reduced the levels of both WT-KRAS and MUT-KRAS in the circIFNGR2 overexpression group (Fig. 5c, e). Furthermore, we subsequently performed western blotting to detect the protein expression level of the corresponding KRAS downstream signaling proteins such as p-ERK and p-AKT. The results showed that the protein expression level of p-ERK and p-AKT was upregulated in the circIFNGR2 overexpressed group of WT-KRAS cells (Fig. 5d), while the protein expression level of p-ERK and p-AKT showed little difference in the MUT-KRAS CRC cells groups (Fig. 5f).

Taken together, albeit miR-30b could downregulate the expression of KRAS, circIFNGR2 could reverse the KRAS-downregulated effect of miR-30b. By directly targeting miR-30b, circIFNGR2 upregulated WT-KRAS and activated its downstream signaling genes in the WT-KRAS CRC cells.

### circIFNGR2 induces cetuximab therapeutic resistance in CRC cells

**Fig. 6:**
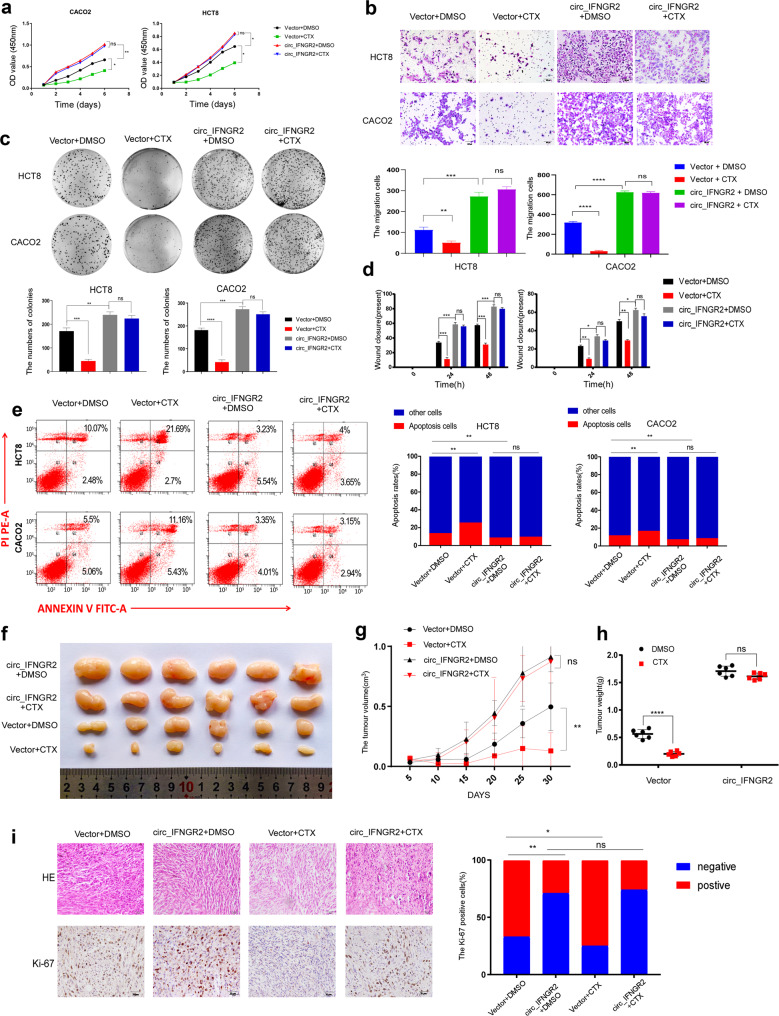
Overexpression of circIFNGR2 lowers the sensitivity of CRC cells to cetuximab. HCT8 and CACO2 cells were divided into four groups, which were respectively treated with vector + DMSO, vector + Cetuximab, circIFNGR2 + DMSO or circIFNGR2 + Cetuximab. **a** CCK8 assay revealed the cell growth ability of the indicated cells. **b** The transwell chamber assay detected the migration ability of the indicated cells. **c** Plate colony formation assay confirmed the proliferation ability of the indicated cells. Representative images(left); Statistical analysis of experimental results(right). **d** Statistical analysis of wound healing assay results for specified cells. **e** Flow cytometry for apoptosis detection of indicated cells (left). Statistical analysis of flow cytometry results (right). **f** The image of the tumor xenograft model (*n* = 5). **g** Statistical analysis of tumor volume at a specific time point for the four designated groups. **h** Statistical analysis of tumor weight among the four designated groups. **i** Immunohistochemical analysis and HE staining of xenograft tumors. The error bars represent the means ± SDs from three independent experiments. *p < 0.05, **p < 0.01, ***p < 0.005.

Cetuximab is a monoclonal antibody that targets EGFR, which can specifically bind to the extracellular EGFR domains expressed on the surface of a variety of cancer cells and competitively block the combination between EGFR and corresponding ligands [31]. Cetuximab can induce tumor cell apoptosis, inhibit cellular processes from the gene level and also combine with the Fc fragment receptor of effector cells such as NK cells to kill tumor cells by antibody-dependent cellular cytotoxicity (ADCC) [31]. Given that circIFNGR2 contributed to properties of cancer cells in CRC, we next examined whether circIFNGR2 led to cetuximab therapeutic resistance (Fig. 6).For the next experiment, we treated colorectal cancer cells with cetuximab and calculated its IC50 (half maximal inhibitory concentration), then constructed cetuximab-resistant CRC cell lines by using concentration gradient method.The CCK8, transwell and colony formation assays showed circIFNGR2 enhanced the proliferation and migration capacities of WT-KRAS CRC cells (Fig. 6a–c) lines and MUT-KRAS CRC cells (Additional file 5: Fig. S4f–h). However, the proliferation and migration of WT-KRAS CRC cells were decreased after treatment with cetuximab while these abilities remained unchanged in circIFNGR2 overexpressed cells regardless of cetuximab treatment (Fig. 6a–c). It was worth noting that both vector and circIFNGR2 overexpressed groups of the KRAS mutant cell lines showed cetuximab chemotherapy resistance (Additional file 5: Fig. S4f–h). The statistical analysis of wound healing assay of WT-KRAS CRC cells (Fig. 6d) further demonstrated that after treatment with cetuximab the migration capacities of cells with no circIFNGR2 transfected were significantly lessened while these capacities of cells in circIFNGR2 overexpression group were not influenced observably. We applied flow cytometry for apoptosis detection of CRC cells to support our hypothesis. As expected, the number of apoptotic cells was increased after treatment with cetuximab in the control group with no circIFNGR2 transfected. However, cetuximab efficacy was nearly eliminated while circIFNGR2 was overexpressed (Fig. 6e).

The following experiments in vivo also consolidate the role of circIFNGR2 playing in the resistance of cetuximab. Xenograft models in nude mice (Fig. 6f–h) demonstrated that circIFNGR2 restrained the cetuximab and led to the rapid growth, larger volume and greater weight of CRC cells, compared to the control groups. Similarly, there was no significant change in the numbers of Ki-67 positive cells from the subcutaneous tumor after the treatment of cetuximab in the circIFNGR2-overexpressing group (Fig. 6i). However, the numbers of Ki-67 positive cells decreased in the control groups. CCK8 assay result shown that the higher concentrations of KRAS led to lower anti-cancer effect of cetuximab. Moreover, CCK8 assay and flow cytometry (Additional file 4: Fig. S3b, c) for apoptosis detection of CRC cells showed that the higher concentrations of KRAS (Additional file 4: Fig. S3a) led to larger resistance of cetuximab. In addition, clinical statistics of WT-KRAS CRC patients with high and low circIFNGR2 expression respectively shown high and low resistance to cetuximab (Additional file 4: Fig. S3d).

Taken together, these results indicated the cetuximab therapeutic resistance in WT-KRAS CRC cells was induced by circIFNGR2 via targeting KRAS.

## Discussion

CRC is one of the most common cancers worldwide, with aberrant regulation of various signaling such as Wnt, Notch and ErbB signaling pathway etc [3]. The epidermal growth factor receptor (EGFR) is one of the members of the ErbB family, involved in nearly all kinds of biological cellular processes. In the presence of ligands such as transforming growth factor alpha (TGF-a), heparin-binding EGF-like growth factor (HB-EGF) and amphiregulin, activated EGFR changes its conformation and combines with the other member of EGFR family to form the dimer, followed by tyrosine autophosphorylation and signaling cascades [32]. One of the crucial downstream pathways of EGFR is the RAS/RAF/MEK/ERK/MAPK pathway in which KRAS plays a significant role [33]. KRAS is a member of the RAS family and it is regulated by GEFs and GAPs. GEFs take part in the conversion of GDP to GTP and lead to the activated state of GTP-bound RAS, in contrast, GAPs induce the hydrolysis of GTP and contribute to the inactivated state of GDP-bound RAS [34]. With the activation of EGFR, KRAS is transiently activated and RAS–GTP dimers recruit RAFs. After MEKs and ERKs are phosphorylated in turn, phosphorylated ERKs relocate to the nucleus, mediating various cellular responses [35–37]. However, the mutated KRAS shows little sensitivity to GAPs and thereby keeps the constantly activated state no matter whether the upstream EGFR is combined with the stimuli [38].

Targeting the EGFR, the monoclonal antibody cetuximab blocks the combination of EGFR and other ligands and thereby suppresses the downstream pathways transduction [5]. Thus, given that cetuximab exhibits promising efficacy in anti-cancer therapy, it has been applied as the first-line treatment of CRC, especially the metastatic CRC [5]. Despite the great clinical efficacy of cetuximab such as the promotion of progression-free survival (PFS) and overall survival (OS), the improvement in the quality of life of patients and the low side effects, the benefits of cetuximab in CRC has been limited due to the chemotherapy resistance [7–9]. The main causes in the cetuximab resistance could be concluded as the following mechanisms. One is the abnormal molecules in the EGFR pathway such as mutations of RAS, BRAF, PIK3CA, PTEN, and TP53; the other is the abnormal activations between the parallel pathways such as the amplification of HER2, HER3 and MET. Besides, other unknown mutations related to dMMR/pMMR or epigenetic instability may also exert influence in the cetuximab resistance [33, 39]. As is mentioned above, mutation of KRAS is considered to be a relatively imperative mechanism. Cetuximab presents relatively great therapeutic effect only among KRAS wide-type CRC patients while the KRAS mutant-type CRC patients show cetuximab resistance [12], for mutant KRAS and downstream pathways are activated and the tumor cell proliferates constantly. What is doubtful and kind of surprising is that a certain number of KRAS wide-type CRC patients are also clinically resistant to cetuximab [40] and the specific mechanisms remain unclear.

Our previous study has verified that miroRNA-30b (miR-30b) directly targeted KRAS, acting as a tumor suppressor in CRC [26]. It was revealed that in CRC tissues, miR-30b inhibited cancer cells cycle progression and the expression of miR-30b was negatively correlated with the KRAS expression. Moreover, miR-30b decreased the expression of genes in downstream pathway of KRAS, indicating that miR-30b could not only reduce the expression of KRAS but also inactivated RAS/RAF/MEK/ERK/MAPK pathway. However, the negative correlation between miR-30b and KRAS in individual CRC samples, which is proven by repeated RT-qPCR experiments, was not that significant. We next wondered why certain samples exhibited the simultaneously high expression of both miR-30b and KRAS and doubted whether other factors were involved to interfere the combination of miR-30b and KRAS, leading to their consistent expression level. On the previous consideration that certain circRNAs probably participate in cetuximab resistance among KRAS wide-type CRC patients, together with the mechanism that circRNAs sponge miRNAs, we wondered whether certain circRNAs could directly target miR-30b and activate KRAS to induce cetuximab resistance in KRAS wide-type CRC patients.

In our research, we used the bioinformatics website starBase to predict that circIFNGR2 could bind to miR-30b. We subsequently examined the expression of circIFNGR2 in both WT-KRAS and MUT-KRAS CRC biopsies and their adjacent normal tissues via RT-qPCR. Results revealed that circIFNGR2 was aberrantly upregulated in CRC tissues as expected and it was correlated with the clinical T stage. Functional experiments were performed to demonstrate that circIFNGR2 overexpression could enhance the proliferation, invasion migration and metastasis capacities of CRC cells.

In order to lucubrate the association of circIFNGR2 and miR-30b, luciferase activity and RT-qPCR revealed the negative correlation of their expression. FISH assay revealed the apparent co-localization of circIFNGR2 and miR-30b in cytoplasm. On top of that, RNA pull-down and RIP assays forcefully, convincingly demonstrated the directly binding of circIFNGR2 and miR-30b. Since circIFNGR2 could sponge miR-30b, we next examined whether it could inhibit miR-30b transcriptional regulatory ability via experiments in vivo and in vitro. Consistent with our expectation, the results strongly confirmed circIFNGR2 could suppressed the anti-cancer effect of miR-30b. Considering that miR-30b targets KRAS, we next wondered whether circIFNGR2 could affect the expression of KRAS by targeting miR-30b. After further verification of the relationship between KRAS and miR-30b by RNA pull-down and RIP assays together with the functional experiments, RT-qPCR and Western blotting assays respectively revealed that circIFNGR2 exhibited antagonism effect in the KRAS gene expression and protein expression against miR-30b. We next further detected the correlation between circIFNGR2 and KRAS including the wild type and mutant type. circIFNGR2 was overexpressed in both wild type and mutant KRAS mutant cancer tissue compared with normal tissue. Furthermore, there was no significant difference in circIFNGR2 expression between wild type and mutant KRAS CRC. Kaplan–Meier survival analysis showed that high expression of circIFNGR2 was tightly related to a poor prognosis of wild type and mutant KRAS CRC patients. The results of western blotting assays further confirmed that circIFNGR2 increased the protein and mRNA levels of both WT-KRAS and MUT-KRAS in CRC cells. Conversely miR-30b reduced the protein and mRNA levels of both WT-KRAS and MUT-KRAS in the circIFNGR2 overexpression group. Given that miR-30b could also silence the RAS/RAF/MEK/ERK/MAPK pathway, it was indicated that as the antagonist of miR-30b, circIFNGR2 could indirectly activate the RAS/RAF/MEK/ERK/MAPK pathway. Thus, we subsequently performed western blotting to detect the protein expression level of the corresponding KRAS downstream signaling proteins such as p-ERK and p-AKT. Results demonstrated that circIFNGR2 upregulated the protein expression level of p-ERK and p-AKT in the WT-KRAS cells, while the protein expression level of p-ERK and p-AKT showed little difference in the MUT-KRAS CRC cells groups. On the basis of these results, finally we unraveled the association among circIFNGR2, miR-30b and cetuximab resistance in WT-KRAS and MUT-KRAS CRC patients. circIFNGR2 enhanced the proliferation and migration capacities of both WT-KRAS and MUT-KRAS CRC cells. In the WT-KRAS CRC patients, circ_ IFNGR2 could upregulate the expression of KRAS, activating the downstream AKT signaling and promoting the cetuximab resistance of CRC cells, while in the MUT-KRAS CRC patients, circ_ IFNGR2 contributed little to the cetuximab resistance for the persistently activated downstream signaling of KRAS. What is more, in the WT-KRAS cell lines, the number of apoptotic cells in the control group increased with the treatment of cetuximab while the circIFNGR2 group shown little efficacy of cetuximab. The further experiments of in WT-KRAS CRC in vivo also demonstrated circIFNGR2 could led to the cetuximab resistance. On top of that, CCK8 assay result showed that the higher concentrations of KRAS led to lower anti-cancer effect of cetuximab.

By and large, our research explored the molecular mechanism of cetuximab resistance in WT-KRAS and MUT-KRAS CRC patients. circIFNGR2 could enhances proliferation and migration of CRC and induces cetuximab resistance. We thought circIFNGR2 could act as a new molecular biomarker for the treatment and diagnosis of CRC patients.

## Conclusion

In summary, a new regulation network of circIFNGR2 was expounded in CRC. Our results demonstrated that circIFNGR2 induces cetuximab resistance in colorectal cancer cells by indirectly regulating target gene KRAS by sponging miR-30b at the post-transcriptional level. It is indicated that circIFNGR2 can be considered as a potential diagnostic biomarker in CRC and inhibition of circIFNGR2 can be a promising therapeutic strategy for malignant CRC patients with cetuximab resistance.

## Supplementary information

Reproducibility checklist

Supplementary Figure 1

Supplementary Figure 2

Supplementary Figure 3

Supplementary Figure 4

Supplementary Figure 5

original data files

table S1

Supplementary figure legend

## Data Availability

All data generated or analyzed during this study are included in this published article and its Additional files.
