# Supplementary figures and images for: CircIFNGR2 enhances proliferation and migration of CRC and induces cetuximab resistance by indirectly targeting KRAS via sponging to MiR-30b
Source: Cell Death Dis. 2023 Jan 13;14(1):24. doi: 10.1038/s41419-022-05536-8 (PMC9839739; doi:10.1038/s41419-022-05536-8)

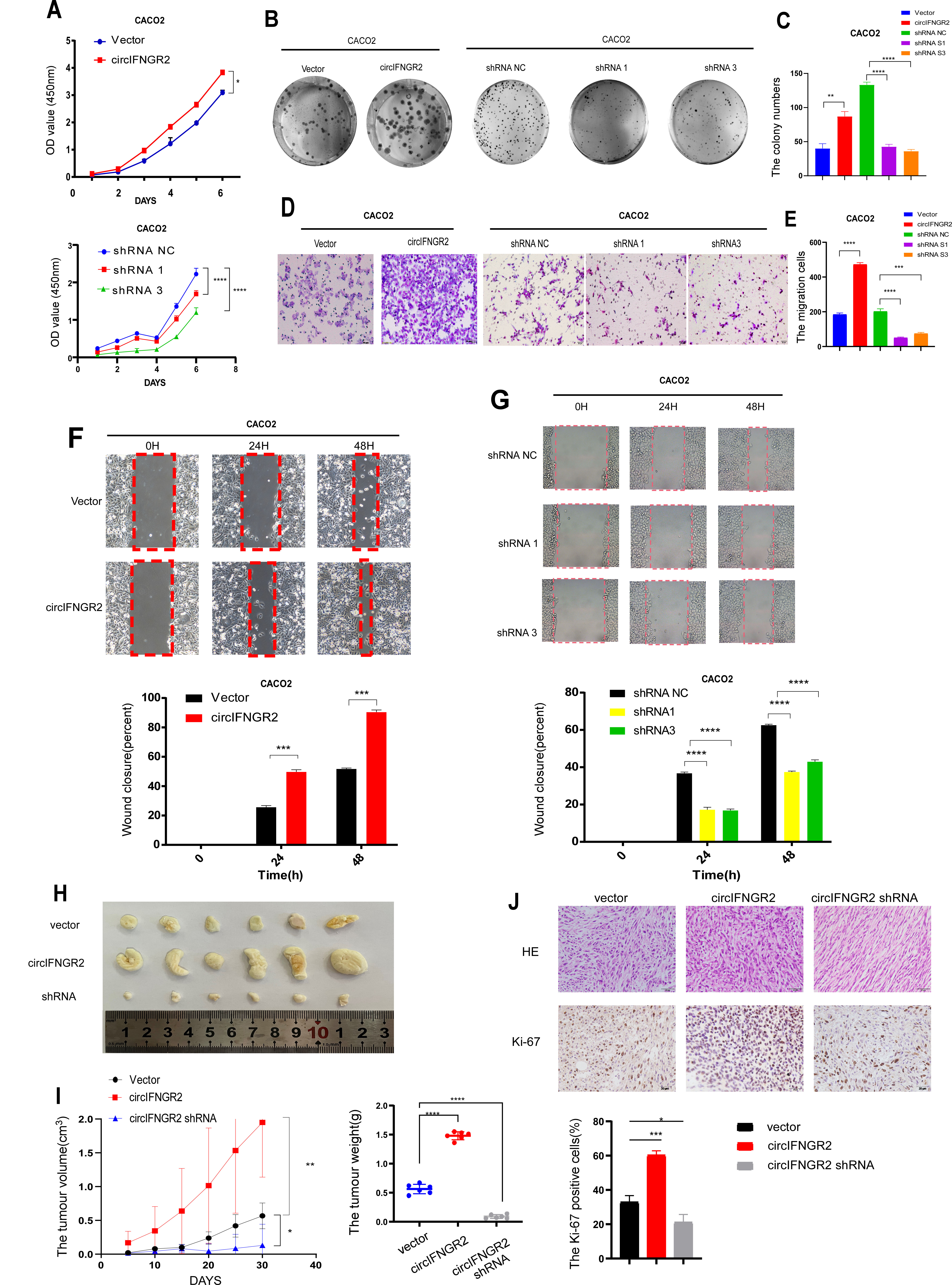

Supplement: Supplementary file 2 — Supplementary Figure 1 [file 41419_2022_5536_MOESM2_ESM.jpg]

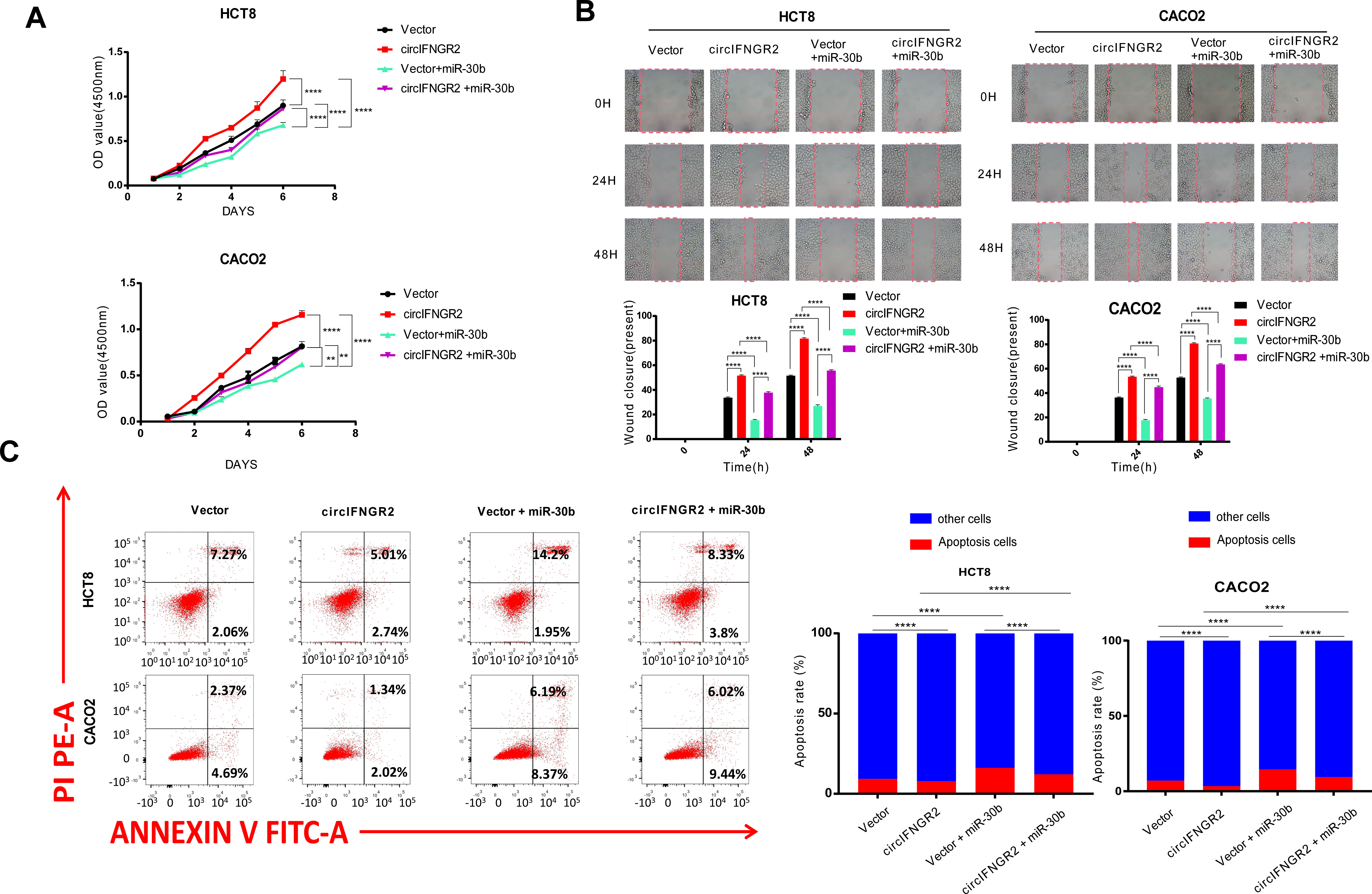

Supplement: Supplementary file 3 — Supplementary Figure 2 [file 41419_2022_5536_MOESM3_ESM.jpg]

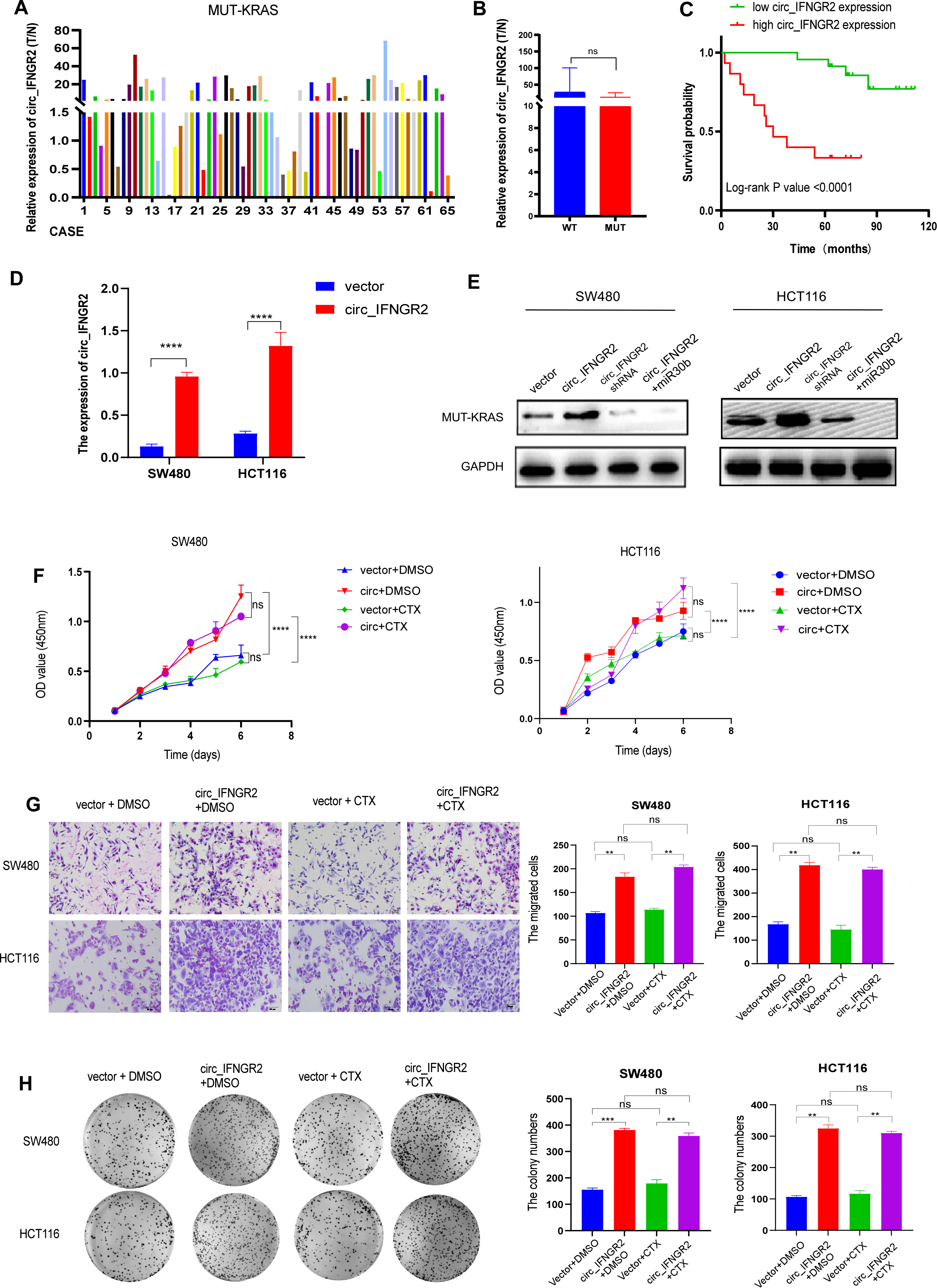

Supplement: Supplementary file 5 — Supplementary Figure 4 [file 41419_2022_5536_MOESM5_ESM.tif]

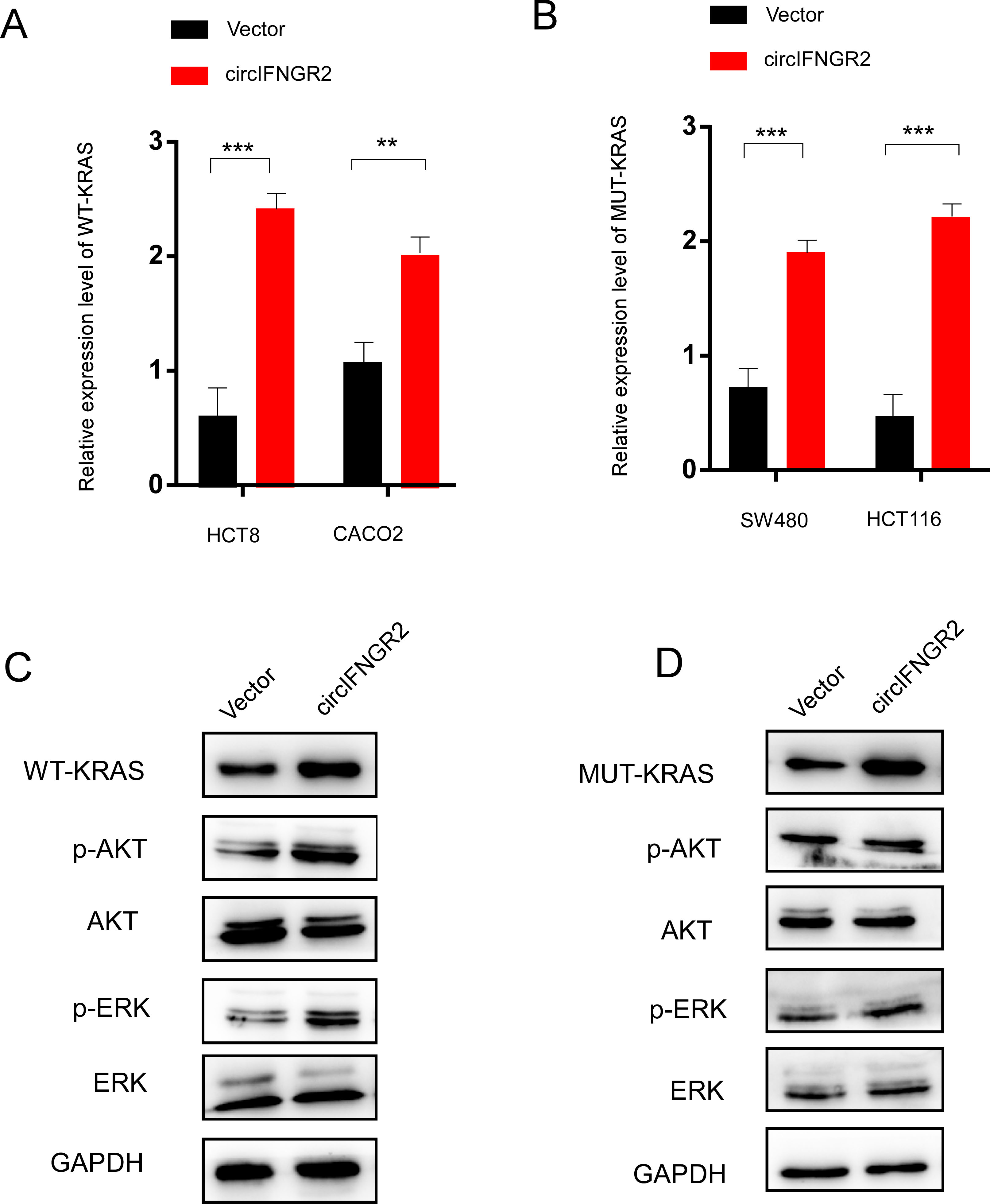

Supplement: Supplementary file 6 — Supplementary Figure 5 [file 41419_2022_5536_MOESM6_ESM.jpg]

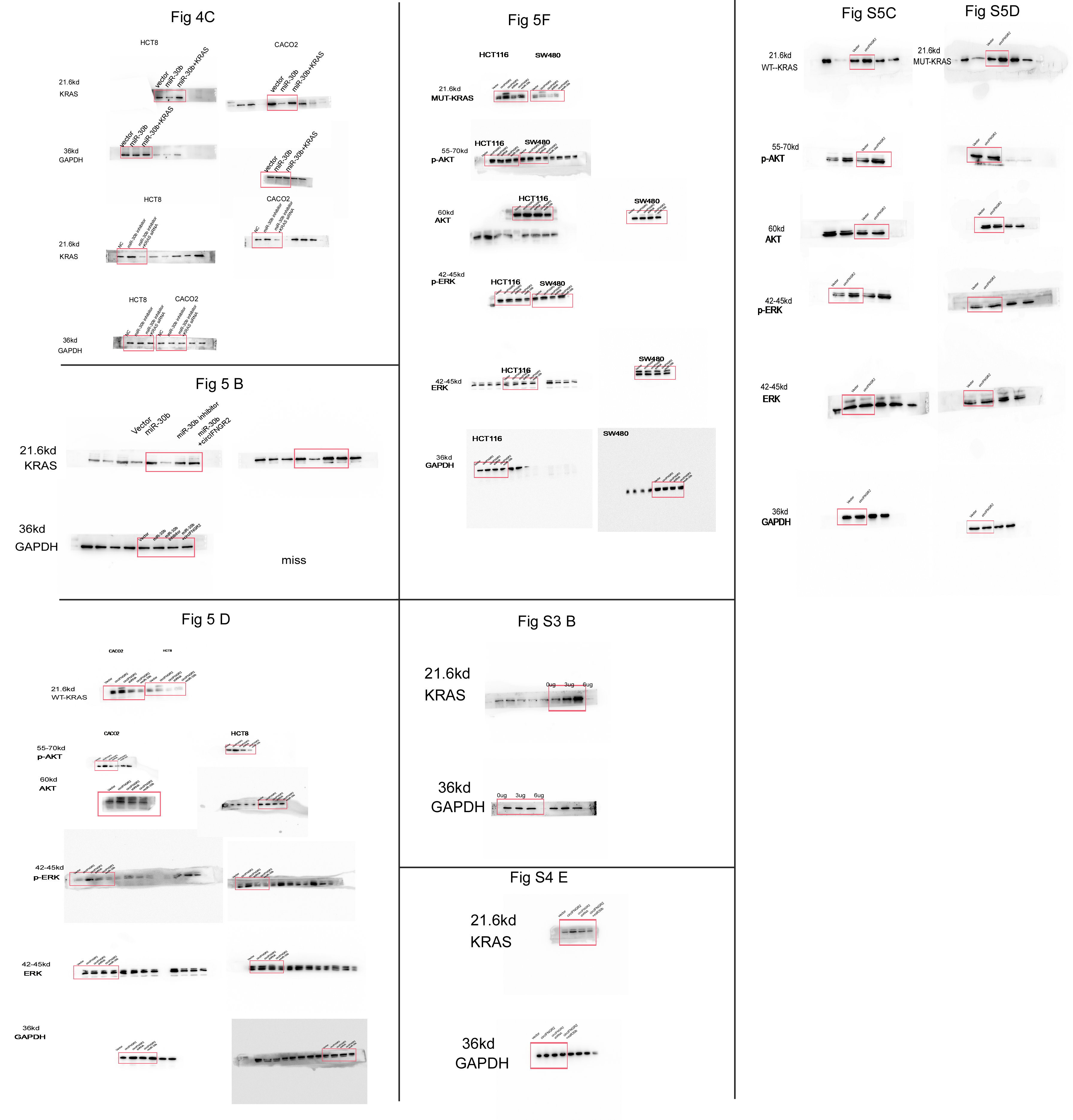

Supplement: Supplementary file 7 — original data files [file 41419_2022_5536_MOESM7_ESM.tif]
