# Supplementary material for: CircIFNGR2 enhances proliferation and migration of CRC and induces cetuximab resistance by indirectly targeting KRAS via sponging to MiR-30b
Source: Cell Death Dis. 2023 Jan 13;14(1):24. doi: 10.1038/s41419-022-05536-8 (PMC9839739; doi:10.1038/s41419-022-05536-8)
Supplement: Supplementary file 8 — table S1 [file 41419_2022_5536_MOESM8_ESM.docx]

Supplementary Table S1

**Primer and probe sequence**

| name | Upper primer（5’-3’） | Down primer（5’-3’） |
| --- | --- | --- |
| GAPDH | GGACCTGACCTGCCGTCTAG | GTAGCCCAGGATGCCCTTGA |
| U6 | CTCGCTTCGGCAGCACA | AACGCTTCACGAATTTGCGT |
| circ_IFNGR2 | GCTTCTGAAAGGGCCTTTGAC | TACGAAACAATGGCAGATGCC |
| KRAS | GACTGAATATAAACTTGTGGTA | GTCCACAAAATGATTCTGA |
| Hsa-miR-30b-5p-probe | AGCTGAGTGTAGGATGTTTAC | |
| Hsa-circ_IFNGR2-probe | CCTTTGACCTCTTCTATCTGTAATGGGA | |
