# Supplementary material for: CircIFNGR2 enhances proliferation and migration of CRC and induces cetuximab resistance by indirectly targeting KRAS via sponging to MiR-30b
Source: Cell Death Dis. 2023 Jan 13;14(1):24. doi: 10.1038/s41419-022-05536-8 (PMC9839739; doi:10.1038/s41419-022-05536-8)
Supplement: Supplementary file 9 — Supplementary figure legend [file 41419_2022_5536_MOESM9_ESM.docx]

**Fig S1. circIFNGR2 affected the proliferation ability of CRC cells**

1. CCK8 assay showed the effects of circIFNGR2 on cell growth

**b.** Representative images of the colony formation assay in the indicated cells.

**c.** Statistical analysis of the colony formation results.

**d.** Representative transwell chamber results comparing the effects of circIFNGR2 on cell migration between the CACO2-vector and CACO2-circIFNGR2 groups (left) and between the CACO2-NC and CACO2-circIFNGR2 shRNA groups (right).

**e.** Statistical analysis of the transwell chamber assay results.

**f, g.** Wound healing assay results showing the differences in migration capacities in the indicated cells at 3 regular intervals (left); statistical analysis of the wound healing assay results (right).

h. Images of the tumor xenograft model.

**j.** Hematoxylin and Eosin (H&E) and Ki-67 immunohistochemical analysis on subcutaneous tumors from mouse tumor xenograft models (upper), statistical analysis of Ki-67 positive cells (lower).

**i.** Comparison of tumor volume and weight of indicated cells.

**Fig S2. MiR-30b suppresses the pro-oncogenic effect of circIFNGR2.**

**a.** CCK8 assay comparing the effects of circIFNGR2 on cell growth between the HCT8-vector, HCT8-circIFNGR2 groups, HCT8-vector + miR-30b and HCT8-circIFNGR2 + miR-30b(left) and between CACO2-vector, CACO2-circIFNGR2 groups, CACO2- vector + miR-30b and CACO2-circIFNGR2 + miR-30b(right).

**b.** Wound healing assay results showing the differences in migration capacities in the indicated cells at 3 regular intervals.

**c.** The apoptosis assay conducted by flow cytometry in the indicated group. Flow cytometry analyses of HCT8 cells and CACO2 treated with 1.0 μM cetuximab for 24 h.

**FigS3. circIFNGR2 induces cetuximab therapeutic resistance in CRC cells**

**a.** Western blot analysis of KRAS in the indicated plasmid-infected cells.

**b.** CCK8 assay comparing the effects of circIFNGR2 on cell growth between the HCT8-0ug + DMSO, HCT8-0ug + CTX, HCT8-3ug + DMSO, HCT8-3ug + CTX, HCT8-6ug + DMSO and HCT8-6ug + CTX.

**c.** The apoptosis assay conducted by flow cytometry in the indicated group. Flow cytometry analyses of HCT8 cells treated with 1.0 μM cetuximab for 24 h.

**d.** Statistical analysis of the clinical correlation between circIFNGR2 expression and resistance to cetuximab of patients from Nanfang Hospital, Guangzhou, Guangdong, China.

**FigS4. The relation between circIFNGR2 and MUT-KRAS**

**a.** RT-qPCR of circIFNGR2 in 65 paired human CRC tissues and the matched adjacent normal tissues.

**b.** The comparison of circIFNGR2 expression level between KRAS wild type and KRAS mutant CRC tissues.

**c.** Kaplan–Meier survival analysis of circIFNGR2 in the MUT-KRAS CRC patients.

**d.** The transfection efficiency of circIFNGR2 (named as circIFNGR2) in SW480 and HCT116 cells was analyzed by RT-qPCR.

**e.** Western blotting was performed to determine the protein expression of MUT-KRAS in the indicated cells

**f.** CCK8 assay revealed the cell growth ability of the indicated cells.

**g.** The transwell chamber assay detected the migration ability of the indicated cells.

**h.** Colony formation assay confirmed the proliferation ability of the indicated cells.

**FigS5. The relation between circIFNGR2 and KRAS including WT-KRAS and MUT-KRAS**

**a, b.** Relative expression of KRAS in the indicated cells.

**c, d.** Relative protein expression of KRAS and its downstream proteins in the indicated cells.
